# Progress in research on and classification of surgical methods of arthroscopic reconstruction of the ACL and ALL using a shared tendon graft through the femoral tunnel

**DOI:** 10.3389/fsurg.2023.1292530

**Published:** 2023-12-22

**Authors:** Ziteng Guo, Fei Liu

**Affiliations:** ^1^ Department of Orthopedics, The First Hospital of Qinhuangdao, Qinhuangdao, China; ^2^ School of Graduate, Hebei Medical University, Shijiazhuang, China

**Keywords:** anterolateral ligament, anterior cruciate ligament, rotatory instability, ligament reconstruction, arthroscopic technique

## Abstract

Anterior cruciate ligament (ACL) tear is a common clinical injury, and ACL reconstruction has reached a very mature stage. However, with the accumulation of cases, scholars have found that isolated ACL reconstruction may not completely solve the problem of knee rotational stability. With the increase in our understanding of knee joint structure, ACL combined with anterolateral ligament (ALL) reconstruction has become accepted by most scholars, and this operation has also achieved good clinical results. At present, there is no unified surgical method for ACL combined with ALL reconstruction. There are differences in bone tunnel location, reconstruction methods, and graft selection. Compared with the independent reconstruction of the ACL and ALL during the operation, shared tendon graft reconstruction of the ACL and ALL has the advantages of preserving tendon and avoiding tunnel convergence. So far, there is no relevant literature summarizing the reconstruction of the ACL and ALL with a shared tendon graft. This paper reviews the anatomic study of the ALL, the study of isometric points, surgical indications, and surgical methods and their classification for shared tendon graft reconstruction of the ACL and ALL.

## 1. Background

The anterior cruciate ligament (ACL) has attracted much attention in the segment of the population with high sporting requirements. According to statistics, the annual incidence of ACL tear is 68.6/100,000, and the incidence in men is significantly higher than that in women (1). However, 10%–20% of patients with ACL reconstruction still have anterior–posterior and rotational instability of the knee (2, 3). There are many factors leading to instability of knee rotation, such as increased tibial posterior inclination, lateral meniscus injury, and lateral complex injury. In 1879, Paul Segond discovered the course of a pearl-like, resistant fiber band and the anatomical structure of the attachment point of the tibia and femur. This ligament showed extreme tension during forced internal rotation of the knee (4). In 2012, Vincent et al. named this ligament the anterolateral ligament (ALL) (5). By 2013, Dr. Carl Claes, a Belgian doctor, had qualitatively and quantitatively studied the course of the ALL and the anatomical structure of the tibia and femur attachment points through autopsy and imaging technology (6). Many scholars began to test the biomechanics of the ALL, attempting to determine the anatomical function of the ALL, the impact of ALL rupture on the knee, and the effect of using various graft sources to reconstruct the ALL. With a heightened understanding of the anatomy and biomechanics of the ALL, ACL combined with ALL reconstruction had come to be widely accepted for the treatment of patients with a high pivot shift test score and ACL reconstruction failure. However, there is no unified surgical method for ACL combined with ALL reconstruction. There are differences in bone tunnel positioning, knee flexion angle during graft fixation, reconstruction methods, and graft fixation methods. Shared tendon graft reconstruction of the ACL and ALL has the advantages of preserving tendon and avoiding tunnel convergence. At present, no scholars have summarized the surgical methods or classification of this operation. This paper reviews the anatomical study of the ALL; the study of isometric points; and the surgical indications, methods, and classification of the joint reconstruction of the ACL and ALL with a shared tendon graft through the femoral tunnel.

## 2. Anatomical structure of the ALL

The anatomy of the ALL remains controversial. In the study of human specimens, the rate of detection of the ALL varies from 12% to 100% (7–11). In most specimens, the ALL can be regarded as a ligament structure. In some cases, when the knee is rotated inward, it may only be observed as a tighter bundle of cystic tissue (12). It is reported that the ALL originates from the femur and inserts into the tibia. When fully extended, its average length is 33–37.9 mm, the average width is 7.4 mm, the average thickness is 2.7 mm, and the average cross-sectional area is 1.54 mm^2^ (13). Thus far, a large number of studies have explored the anatomical attachment points of the ALL, and the tibial attachment point of the ALL is less controversial. According to Parker and Smith, the ALL stops between the Gerdy tubercle and the fibular head; in most cases, it ends at the midpoint between the Gerdy tubercle and the fibular head, and approximately 30.2% of ALL tibial stops are close to the fibular head (14). When reconstructing the ALL, clinicians often choose the midpoint between the Gerdy nodule and the fibular head. However, experts’ descriptions of the femoral side are quite different. Claes et al. believed that the femoral attachment point of the ALL was located slightly in front of the attachment point of the lateral collateral ligament (6). The following year, Helito et al. studied the imaging landmarks of the ALL and claimed that on the lateral radiograph of the knee joint, the femoral attachment point of the ALL was approximately 47% of the anteroposterior diameter of the lateral condyle (from the front), 3.7 mm below the Blumensaat line. The tibial attachment point of the ALL was approximately 7.0 mm below the joint line and 53.2% of the front and rear diameters of the tibial plateau (from the front) (15). Daggett et al. and Dodds et al. found that the femoral attachment point of the ALL was located at the posterior and proximal part of the femoral lateral epicondyle or directly on the femoral lateral epicondyle (9, 16). Many studies of different anatomical structures show that the morphology of the ALL is not completely consistent, and there are some variations in the starting and ending points and course of the ALL. In 2018, Olewnik et al. studied the anatomy of lower limb specimens and divided them into five types according to the morphology of the ALL (17). In Type I, the ALL starts from the lateral femoral condyle, extends in front of the proximal lateral collateral ligaments (LCL), down and parallel to the LCL, and ends behind the Gerdy tubercle of the tibia. In Type II, the ALL starts from the lateral condyle of the femur, behind the proximal end of the LCL, crosses the LCL obliquely forward and downward, and ends behind the Gerdy tubercle of the tibia. In Type III, the ALL starts from the lateral femoral condyle, the posterior LCL, and the lateral posterior joint capsule, showing a wide fan shape. In Type IV, the ALL is characterized by double bundles, both of which start from the lateral femoral condyle in front of the LCL, with the front bundle ending behind the Gerdy tubercle and the rear bundle ending at the deep fascia. In Type V, the ALL starts from the LCL and ends behind the Gerdy tubercle of the tibia. In general, the variation in the femoral attachment point is high, and the variation in the tibial attachment point of the ALL is low; this is mostly located in the middle of the Gerdy tubercle and the fibular head.

## 3. Biomechanics of the ALL

In terms of biomechanics, current studies show that the ultimate load of the ALL is 50–205 N, the average stiffness is 20–42 N/mm, and the average ultimate strain is 36% (18). These results emanate from differing descriptions of the anatomy of the ALL. Zens et al. separated an isolated ALL structure in their experiment and measured an ultimate strength for it of only 50 N (19). The reason why the limit load of the ALL varies greatly in different studies may be that the structure of the ALL is not completely separated and the capsulo-osseous layer of the iliotibial band is included in the measurement (20). Multiple studies have focused on the biomechanics of ALL- and ACL-deficient knees. Some studies have shown that the ALL resists internal rotation when knee flexion exceeds 35° (21). Other studies have shown that when the knee joint is subjected to an external force of 30° bending, the forward translation of ALL-excised knees is significantly more than that of ALL-intact knees (22). However, Rasmussen et al. found that even when the knee is fully extended, the tibial axis plane translation and internal rotation of knees with ALL and ACL defects are significantly increased (23). This could explain why some patients still produce positive pivot shift test results after isolated ACL reconstruction. The current literature shows that the ALL may be an asymmetric structure that plays a very important role in resisting internal rotation, especially when the knee is bent.

## 4. Research on the isometric points of the ALL

The ALL has no obvious isometry during knee joint movement, and its structure is difficult to replicate due to its tissue structure, viscoelasticity, tubular insertion on the femoral side and flake insertion on the tibial side. Determining the most appropriate isometric point is the key to the success of all reconstructions. As initially described by Claes et al., ALL length was found to increase from 32.8 ± 2.5 mm at 0° to 48.5 ± 4.6 mm at 90° with an increase in buckling (6, 24). Subsequently, Helito et al. evaluated the length and isometric pattern of the ALL by computed tomography and found that its length increased by 16.7% on average from full extension to 90° flexion. The increase in length in moving from 60° to 90° is greater than that occurring in moving from 0° to 30° and from 30° to 60°. The length of the ALL increases with the degree of buckling. This study showed that the tension also increases with an increase in buckling (25). Zens et al. fixed a highly elastic capacitive strain gauge at the femoral and tibial insertion of the ALL, recorded ALL length when the knee joint specimen was passively moving at different angles, and compared it with ALL length at the 0°-extension neutral position (26). The results showed that the length of all joints increased with an increase in the flexion angle, and the maximum length could be observed at 90° flexion of the knee joint. This suggests that for ALL reconstruction, the tension and fixation of the graft should be performed near 90° flexion because the tension of the graft near extension may lead to excessive ligament strain and increase knee flexion. However, the research results of Dodds et al. were the opposite (16). They used linear variable displacement sensors to measure the change in ALL length from the straight position to 90° flexion of the knee joint and found that the ALL was nearly isometric from 0° to 60°, while the ligaments gradually relaxed over 60°. The reason is that scholars have chosen different sites to simulate the changes in knee flexion and extension length of the ALL, and there are few experimental samples. During the ALL reconstruction process, if the fixed point is not at the isometric point, the length and tension of the reconstructed ligament will significantly change with joint activity, leading to complications such as limited knee joint mobility or graft tear or relaxation, resulting in poor control of knee joint rotational stability and postoperative residual axial shift. Therefore, determining the most suitable isometric point is the key to ALL reconstruction surgery. Similar to the functional reconstruction of lateral extra-articular tenodesis (LET), scholars are also seeking sites for ALL functional reconstruction. However, Kittl et al. established a knee extension device by using a suspended weight and pulley system, paired to measure different combinations of femoral and tibial values, and found that no pair could achieve perfect isometric points (27). Kernkamp et al. conducted isometric studies in 2017 using MRI and biplane x-ray techniques, combined with ALL anatomical attachment points, and claimed that the most isometric attachment point on the femur should be located posterior and distal to the femoral attachment point of the lateral collateral ligament (28). Based on different insights into the anatomy of the ALL, studies by Kittl et al. and Imbert et al. defined the best compromise between the anatomical structure of the ALL and the ideal tunnel position for ALL reconstruction, which tends to be isometric to the ligaments: the outer opening of the femoral tunnel is located on the posterior superior side of the lateral epicondyle, and the tunnel on the tibial side is located on a line between the Gerdy nodule and the fibular head (27, 29). More research results are needed to provide reliable evidence on the isometric points of the ALL.

## 5. Indications for reconstruction of the ACL and ALL

With the deepening understanding of the ALL of the knee joint, the indications and techniques for surgery for reconstruction of the ACL and ALL of the knee joint are also constantly changing. In the consensus released by the ALL expert group in 2017, it was mentioned that routine ALL reconstruction should not be performed for patients undergoing ACL reconstruction, but when the patient meets one decisive criterion or two secondary criteria, ACL combined with ALL reconstruction should be considered (30). The decisive criteria for ACL and ALL reconstruction indications are as follows: significant anterior lateral rotation instability in ACL revision cases; axial shift test II–III degrees before the initial Anterior cruciate ligament reconstruction (ACLR) surgery; imaging findings suggesting Segond fracture; systemic multiple ligament relaxation (Beighton score ≥4) or knee joint flexion (>10°); or being a sports participant engaged in activities involving knee joint rotation. The secondary criteria are: the presence of damage to the contralateral ACL; a difference greater than 7 mm between the two knees in the Rahman test; MRI scan revealing a deep lateral femoral condylar notch sign; or age <25 years old. Many scholars believe that a preoperative high-degree axial shift test is a very important surgical indication for patients. The axial shift test is widely used to evaluate the dynamic rotational stability of the knee joint (31). The most commonly used method is to bend the knee from 0° (fully extended) to 90° while applying external rotational stress to the tibia. If the knee is subjected to external rotational stress below the femoral condyle and the tibia is rapidly anterior subluxate at 20°–30° flexion, the test results are positive (32). The International Knee Documentation Committee (IKDC) classification defines the following grading system for pathological movements observed in pivot shift tests: 0 (normal), 1 (sliding), 2 (clumsy), or 3 (locked subluxation) (33). From a functional perspective, this is a reproduction of the phenomenon of knee collapse when the anterior cruciate ligament is broken. In theory, the pivot shift test is an ideal test for dynamically evaluating the status of knee ligaments. However, the main challenge in clinical practice is that muscle resistance can inhibit central shift, especially in patients with knee joint soft tissue injury and swelling. Therefore, many studies have been conducted under anesthesia rather than when the patient is awake, and it is inevitable that subjective factors in physicians’ judgments also affect the experimental results. Recently, several tools have been developed to quantitatively evaluate knee joint rotational relaxation, such as surgical navigation, electromagnetic sensor systems, and triaxial accelerometers (34). Among them, the triaxial accelerometer is a small, non-invasive system that is easy to apply in clinical practice. It evaluates the phenomenon of pivot shift by measuring tibial acceleration, which more accurately quantifies the axial displacement experiment, making the diagnostic axial displacement test more standardized (35). In terms of imaging diagnosis, the reference value for ALL injury in x-ray films is limited, and only Segond fracture can indirectly determine ALL fracture. MRI mainly diagnoses ALL injury in coronal and T2 pressure lipograms (36). However, the accuracy of knee MRI is lower in identifying the anterolateral structure of the knee than in identifying the posterolateral structure (37). According to research by Castelli et al., the ALL is not always visible on MRI (38). Ultrasound examination is a reliable method for evaluating the anterior lateral ligament (39). Ultrasound can check the integrity of the ALL under static conditions and can also dynamically check the ALL using Doppler mode. The specific method is as follows: hold the patient's foot and rotate the knee inward to apply tension to the ALL. For patients with an intact ALL, this rotation will block the blood flow of the lateral inferior genicular artery (LIGA). If the patient's ALL is broken, it will not compress the LIGA, and no break in LIGA blood flow will be observed in Doppler mode (40). This measurement method causes minimal pain stimulation for patients and is widely accepted by patients. At present, further exploration is still underway for imaging diagnosis of ALL rupture, and more research results are needed to provide reliable evidence.

## 6. Graft types for ACL and ALL reconstruction

Before the ALL structure was recognized by most scholars, anterior lateral structural reconstruction was the surgical method chosen by most physicians to achieve rotational stability of the knee joint. Lemaire chose to use an 18-cm-long and 1-cm-wide iliotibial tract for functional reconstruction of the anterolateral structure (Lemaire procedure) (41). Subsequently, an improved Lemaire procedure was performed to obtain a 1-cm-wide graft from the middle of the iliotibial tract and preserve its distal attachment point to the Gerdy nodule (42). The MacIntosh procedure involves cutting the iliotibial tract from the middle and preserving its attachment to the Gerdy nodule (43). In 2014, Claes et al. presented the latest research on the ALL, which sparked scholars’ interest in ALL reconstruction (24). Scholars choose different types of tendon grafts, which are mainly divided into two categories: autologous grafts and allogeneic grafts. Shared tendon graft reconstruction of the ACL and ALL often uses a single femoral tunnel. The outer opening of the tunnel is the starting point of the ALL femur, and the inner opening is the attachment point of the ACL femur. Autotransplantation of hamstring and gracilis tendons is a commonly used autologous graft. Helito et al. used a triple-folded semitendinosus muscle and a single-stranded gracilis muscle to reconstruct the ALL and ACL. The semitendinosus was formed into four strands for ACL reconstruction (44). Saithna et al. harvested the semitendinosus and gracilis tendons using an open-ended tendon stripper, ensuring that they were not detached from the tibia at this stage. The semitendinosus tendon was folded into three strands and woven into one end of the gracilis muscle to form a bundle of grafts. Four strands of the graft were used for single-bundle ACL reconstruction, and one strand was used for double-bundle ALL reconstruction after penetrating the femur (45). Some scholars have also used the iliotibial bundle to reconstruct the ALL and ACL. Lutz et al. used the iliotibial bundle for minimally invasive reconstruction of the ALL and ACL (46). They obtained the central part of the iliotibial bundle, making the graft about 20 cm long, 1 cm wide at the distal end, and 3 cm wide at the proximal end, while preserving the tibial insertion point of the ligament. In addition, the peroneus longus muscle can also be used for ACL combined with ALL reconstruction. Escudeiro de Oliveira et al. reconstructed the ALL with an ipsilateral peroneus longus tendon graft and the ACL with a five-fold graft consisting of a double-bundle semitendinosus tendon, a double-bundle gracilis tendon, and a single-bundle peroneus longus tendon (47). Josipović et al. presented a technique of Anterior lateral ligament reconstruction (ALLR) using the ipsilateral plantaris longus tendon. A quintuple graft composed of a three-strand semitendinosus tendon and a two-strand gracilis tendon (used for ACLR) and a two-strand plantaris longus graft (substituted for the ALL), sutured to the quintuple graft, has also achieved good clinical results (48). Allografts can be divided into allografts and synthetic grafts. Some authors recommend the use of allografts in ALL reconstruction, even in the initial operation, emphasizing the advantages of the incidence rate of donor free sites and the availability of larger and longer grafts (49). However, allogeneic grafts are mainly used for revision surgery, where it may not be possible to obtain a sufficient number of autologous grafts (50). The synthetic graft material is polyester tape. Wagih and Elguindy reported an ALL reconstruction technique using polyester tape (51), but the report did not provide information on postoperative outcomes and prognosis or on the rate of ligament re-tearing. Polyester tape reconstruction of the ALL may be worthy of further research (Table 1).

**Table 1:** Graft choices and fixation methods.

Reference	Year	Material	Graft type	Knee flexion/rotation
Lemaire (41)	1980	ITB	Autologous graft	30° flexion/external rotation
Helito (44)	2015	Gracilis–semitendinosus	Autologous graft	60°–90° of flexion
Sonnery (30)	2016	Gracilis	Autologous graft	Full extension/neutral rotation
Lutz (46)	2016	ITB	Autologous graft	30°–90° of flexion
Wagih (51)	2016	Polyester tape	Allogeneic grafts	30° flexion
Oliveira (47)	2021	Peroneus longus	Autologous graft	30° flexion

ITB, The iliotibial band.

## 7. Surgical methods and classification of ACL combined with ALL reconstruction

**Figure 1:**
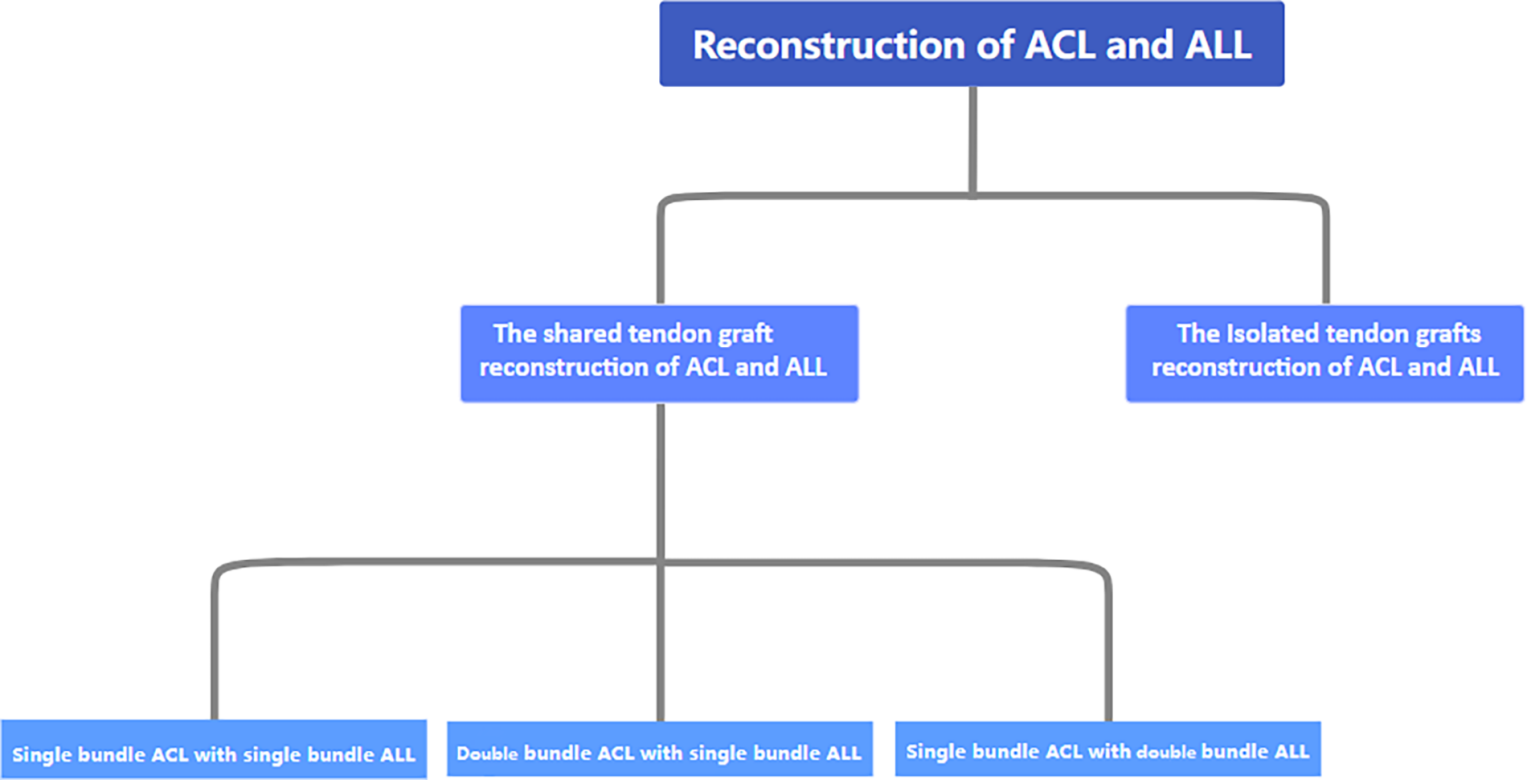
Classification of surgeries for ACL combined with ALL reconstruction.

Recently, ACL and ALL combination reconstruction surgery has received increasing attention from scholars. At present, autologous transplantation of the hamstring muscle, single-bundle reconstruction, an anterior internal portal for femoral tunnel drilling, a cortical suspension system for femoral fixation, and a compression system for tibial fixation represent the current standards for ACLR in the orthopedic community (52). For the reconstruction of the ALL, scholars tend to prefer anatomical reconstruction. Because of the fact that different anatomical points are studied by different scholars, different physician groups choose different reconstruction sites. The common choices for clinical physicians to focus on in terms of the construction site of the ALL femur are the posterior and proximal ends of the external epicondyle of the femur, the proximal and posterior ends of the lateral collateral ligament insertion point, and the anterior side of the femoral attachment point of the external epicondyle and lateral collateral ligament of the femur (44, 50, 53). Most of the tibial lateral insertion points are located near the line connecting the Gerdy and the fibular head. The surgical methods and graft fixation methods for ACL combined with ALL reconstruction also vary. Overall, they can be divided into single-bundle ACL reconstruction combined with single-bundle ALL reconstruction, double-bundle ACL reconstruction combined with single-bundle ALL reconstruction, and single-bundle ACL reconstruction combined with double-bundle ALL reconstruction (Figure 1).

### 7.1. Single-bundle ACL reconstruction combined with single-bundle ALL reconstruction

**Figure 2:**
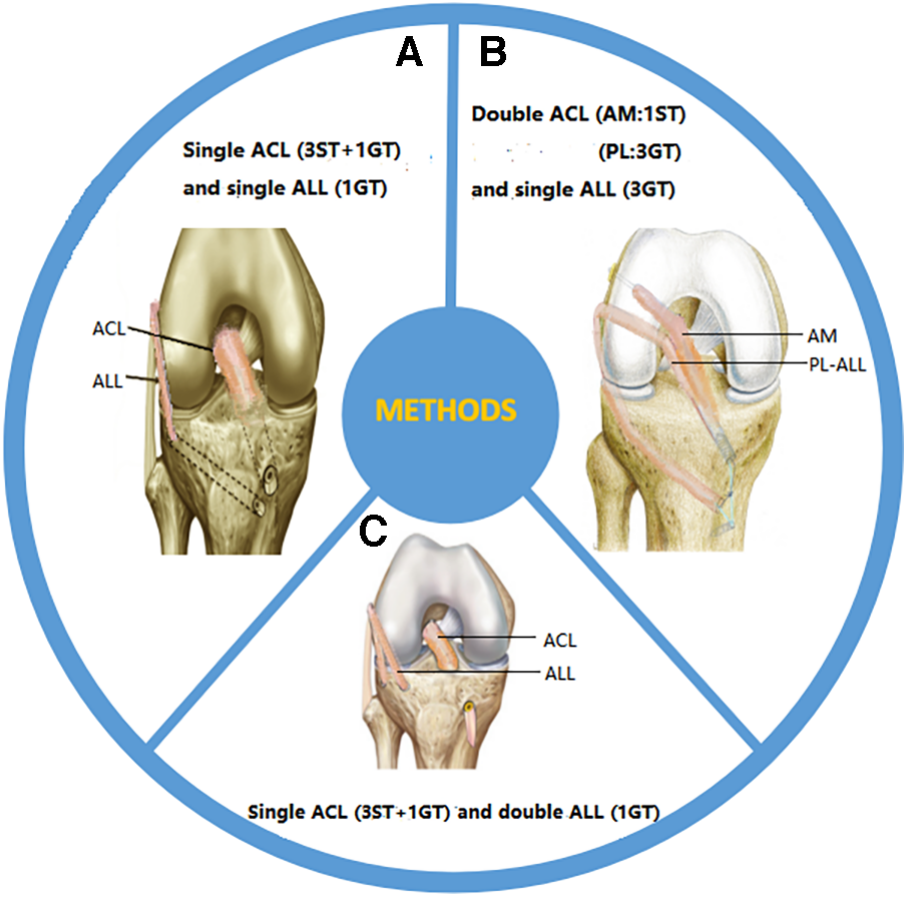
Surgical methods for reconstruction: (**A**) single-bundle ACL reconstruction combined with single-bundle ALL reconstruction; (**B**) double-bundle ACL reconstruction combined with single-bundle ALL reconstruction; (**C**) single-bundle ACL reconstruction combined with dual-bundle ALL reconstruction. ST, semitendinosus tendon; GT, gracilis tendon.

Helito et al. weaved the semitendinosus tendon into a three-strand graft and the gracilis tendon into a single-strand graft. These two types of grafts were stitched together to produce four strands of ACL grafts and one strand of ALL grafts. After the graft penetrated through the tibial tunnel and was used to reconstruct the ALL with a four-strand graft in the joint, the remaining single-strand gracilis muscle graft penetrated through the lateral femoral condyle and was fixed below the iliotibial tract at the ALL tibial insertion point. The femoral and tibial insertion points of the ALL were fixed with absorbable interference screws (54) (Figure 2A). Subsequently, Oliveira et al. applied the same fixation method in clinical practice (47). Despite differences in the transplant sites that they chose, all of them achieved good results. Another reconstruction method is to pull the graft into the joint through the femoral tunnel. After the ACL reconstruction is completed, the remaining graft is pulled out from the external opening of the tibia and fixed to the femur through the lower part of the iliotibial tract (55). The difference between these two methods lies in the different order in which the graft is pulled into the tunnel. So far, no comparison has been made between the clinical effects and graft stability of the two surgical methods, and further research is needed.

### 7.2. Double-bundle ACL reconstruction combined with single-bundle ALL reconstruction

Mediavilla et al. used the gracilis muscle to weave three strands (approximately 7–9 mm) as grafts for the posterior lateral (PL) bundle of the ACL and ALL, and also used the semitendinosus to weave a single strand (approximately 4.5 mm) as grafts for the anterior medial (AM) bundle of the ACL. The PL-ALL graft was pulled into the joint using a traction line from the entrance point of the tibial tunnel, following which the graft entered the PL tunnel. After PL reconstruction was completed, the ALL was reconstructed by leaving the lateral femoral cortex. One end of the graft was retained at the ACL tibial insertion point, while the other end was fixed with a degradable screw at the ALL tibial insertion point. The AM graft was pulled into the joint through the external opening of the tibia and connected to the femoral cortex using an Endo Button suspension device, and the soft tissue graft was accurately positioned in the femoral tunnel (56). Mao et al. cut the ipsilateral hamstring tendon and fibula longus tendon to prepare AM and PL-ALL grafts, respectively. The femoral side of the AM graft was fixed with Endo Button suspension, and the PL-ALL graft was fixed with one compression screw at the external opening of the femoral tunnel. When the graft sutures were anchored to the tibial screw, the AM and PL-ALL grafts were fixed with the fixation screw and absorbable interface compression screw at the tibial side (57) (Figure 2B). In addition, Chiu et al. suggested using suture strips to enhance the internal stent during tendon transplantation in order to protect newly reconstructed ligaments during the rehabilitation process. They combined double-bundle ACL reconstruction with single-bundle ALL reconstruction with autologous hamstring muscle grafts and internal stents, using button suspension fixation devices and aperture screws (58). This technology reduces residual anterior and rotational instability after ACL and ALL reconstruction, achieving good clinical results.

### 7.3. Single-bundle ACL reconstruction combined with dual-bundle ALL reconstruction

Double-bundle ALL reconstruction restores the true anatomy of the tibial insertion point on the ALL side and has received widespread attention from scholars. Sonnery-Cottet et al. obtained the semitendinosus tendon and gracilis tendon as joint reconstruction grafts. The ACL graft consisted of three semitendinosus tendons and an additional gracilis tendon, while the ALL graft consisted of a Y-shaped circular gracilis tendon. The proximal drilling site of the tibial ALL was located at the Segond fracture site (the tibial footprint passing through the ALL), while the distal drilling site was located in front of and below the site. By using traction sutures on the graft, the combined graft was passed proximally through the knee. After the ACL graft was completed, the ALL graft was pulled out through the outer opening of the femoral tunnel and pulled into the outer opening of the tibia (Segond fracture site). The ALL graft was fixed at the outer opening of the femur through a V-shaped tibial tunnel and threaded out from the inner opening of the tibia (59) (Figure 2C). Mogos et al. applied the same fixation method, but the difference lies in the two external tibial openings used to prepare the V-shaped tibial tunnel. One was located at the horizontal plane of the Gerdy nodule, and the other was located in the middle between the Gerdy nodule and the tip of the fibular head (60). They believe that such tibial sites are more in line with the anatomical attachment point on the tibial side of the ALL. Another fixation method has been proposed by Zein et al., who fixed the double-bundle ALL at the same tibial site (the midpoint between the fibular head and Gerdy's nodule, 10 mm from the joint line) when reconstructing the ALL. They created four strands of ACL using semitendinosus and gracilis tendons. Subsequently, the four strands of semitendinosus and gracilis tendons were divided into two separate double-bundle ALL strands. After completion of the ACL reconstruction, the graft penetrated through the lateral condyle of the femur. An ALL through-tunnel was created over each guide pin with a 6-mm cannulated drill bit. A bone bridge was left on the medial tibial cortex between the two tunnels, over which the Ethibond strands attached to the two ALL bundles were tied for fixation. A wire loop was used to shuttle each bundle of the graft into its corresponding tunnel. The Ethibond strands of both bundles of the ALL were tied over the bone bridge on the medial tibial cortex (61). The above surgical methods have achieved good clinical efficacy, but currently, there is no literature comparing the advantages and disadvantages of these surgical methods.

## 8. Tunnel convergence of grafts in ACL and ALL reconstruction

When reconstructing the ACL and ALL with two bundles of grafts, there is a risk of convergence in the femoral-side graft tunnel due to the proximity of the anterior cruciate ligament tunnel to the origin of the ALL. Smeets et al. observed a high risk of tunnel convergence by drilling anatomical ACL femoral tunnels and ALL tunnels in different directions on fresh frozen knee joint cadavers, and noted that tunnel fusion can endanger ACL reconstruction and fixation (62). Rosenstiel et al. combined three-dimensional (3D) CT reconstruction images reconstructed from the ACL with virtual ALL reconstruction to simulate potential tunnel collisions in the femoral ACL tunnel. They found that when the tunnel is drilled at 0° in the axial plane, the risk of tunnel collisions significantly increases, and potential damage to the reconstructed ACL femoral attachment may also occur (63). However, there is no consensus among scholars on the angle of femoral drilling to avoid tunnel collisions. There have been reports of drilling at 0° axial and −40° coronal, 40° perpendicular to the femoral anatomical axis, 30° anterior to the axial plane, and 30°proximal to the coronal plane. Shared tendon graft reconstruction of the ACL and ALL can solve the problem of graft tunnel convergence, mainly because the graft of a single-bundle ACL combined with single-bundle ALL reconstruction and a single-bundle ACL combined with double-bundle ALL reconstruction runs through the femoral tunnel, allowing the ACL and ALL to share the same tendon and tunnel without the risk of tunnel convergence. However, in double-bundle ACL reconstruction combined with single-bundle ALL reconstruction, the PL and ALL share a tendon bundle, while AM alone has a tendon bundle. The problem of ligament convergence may occur in the AM and ALL bundles. In 2021, research by Kawanishi et al. showed that when facing the issue of femoral tunnel collision between ACL and ALL tunnels, AM drilling at 120° and PL drilling at >135° knee flexion, combined with ALL drilling at 30° coronal angle and >45° axial angle, may reduce this risk (64). However, some studies have investigated the relationship between the position of the femoral tunnel exit and the lateral anatomical structures (such as the posterior femoral cortex, gastrocnemius appendage, popliteal tendon, and capsule) during the process of ACL femoral drilling through the anterior medial portal. The conclusion drawn from these studies by Osaki et al. and Farrow and Parker is that it is more advisable to drill holes with the knees bent to 135° instead of 120° to avoid posterior burst and the formation of tunnel exits under soft tissue (65, 66). This is at variance with the conclusions of Kawanishi et al.. Therefore, further research is needed on the safest femoral drilling procedure in the process of dual-bundle reconstruction of the ACL and ALL to prevent femoral tunnel collisions, including all lateral anatomical structural injuries.

## 9. Results of ACL combined with ALL reconstruction

In theory, it is believed that combined surgery can reduce the stress on ACL grafts and protect them. Previous biomechanical studies have shown that additional ALL reconstruction/repair surgery should always be considered in patients with significant ALL tearing prior to surgery (38). Sonnery-Cottet et al. first reported on following up 92 patients with combined reconstruction of the ACL and ALL for more than 2 years in 2015 and found that in the last follow-up, all patients had normal knee joint mobility and a significantly improved Lysholm score, subjective IKDC score, and objective IKDC score (59). The Tegner activity scale (TAS) grade score decreased compared with the preoperative score, and there was a significant reduction in the average anteroposterior shift of the knee joint compared with that observed in the preoperative period. The final Knee injury and Osteoarthritis Outcome Score (KOOS) score reached 88 points, and 71.1% of patients recovered to preoperative activity levels after surgery, indicating that ACL combined with ALL reconstruction can significantly improve the stability of the knee joint. Two years later, they conducted a prospective controlled study on combined reconstruction and found that, compared with single ACL reconstruction, ACL combined with ALL reconstruction significantly reduced the tear rate of grafts after ACL reconstruction. A total of 93% of patients had recovered their motor function at the last follow-up, and 64.6% of patients returned to their preinjury self-described motor activity levels (67). A recent meta-analysis showed that compared with isolated ACLR, patients with ACL combined with ALL reconstruction showed significant improvement in postoperative functional score (mean follow-up time >1 year) and knee anteroposterior stability (68). For populations with high demands for exercise, ACL reconstruction combined with ALL is more suitable. Hamido et al. followed 102 athletes for an average follow-up time of 60 (55–65) months, further confirming that ACL combined with ALL reconstruction is significantly superior to isolated ACL reconstruction in terms of anteroposterior stability and IKDC objective score (69). Therefore, combined reconstruction of the ACL and ALL may reduce the rate of graft tear, improve the rotational stability of the knee joint, and ultimately restore the patient to their preinjury level of motion, which is also our expectation. In addition, compared with isolated reconstruction of the ACL and ALL, shared tendon graft reconstruction of the ACL and ALL using the same graft through the femoral tunnel also has additional advantages. This surgical method not only saves tendons, but also provides continuity in the reconstructed ligaments of the ACL and ALL, making the reconstruction more stable and reducing the number of rivets used, as well as reducing the risk of collisions between the femoral fossa and the anterior cruciate ligament canal of the femur. However, Schon et al. conducted anatomical, reconstructive, and biomechanical studies on 10 samples and found that although the anatomical reconstruction of the ALL combined with the ACL can reduce rotational relaxation when knee flexion exceeds 30°, it can restore knee stability while also causing excessive limitation of normal knee movement (70). Although this study was conducted on specimens, the results still have certain guiding significance and are worthy of deep consideration. The clinical efficacy of ALL reconstruction still needs more postoperative follow-up and further clinical research for confirmation.

## 10. Summary and prospects

Arthroscopic ACL reconstruction is widely used internationally as the gold standard for the treatment of ACL fracture. Despite the successful clinical application of this technique, rotational relaxation and osteoarthritis still occur after surgery. According to Monaco, lesions in the anterolateral structure may be associated with pivot displacement. Therefore, it is more correct and biomechanically effective to evaluate and repair the anterior and lateral structures of the knee during ACL surgery (71). Zaffagnini et al. and Asai et al. quantified the effect of the ALL on the armature displacement of the knee joint using one and two measurement worksheets, respectively, and demonstrated that the ALL plays an important role in maintaining the rotational stability of the knee joint (72, 73). In clinical practice, ACL reconstruction combined with anterior lateral structural reinforcement or reconstruction is significantly superior to isolated ACL reconstruction in terms of postoperative rotational stability, loss of efficiency, and rate of return to motion of the knee joint. However, due to the inconsistent research results on the anatomy of the ALL, there is a lack of uniformity in the corresponding reconstruction techniques. Further anatomical and biomechanical research on the ALL is needed, which will help improve the level of reconstruction techniques. More long-term and high-level clinical follow-up studies are also necessary to clarify whether ACL combined with ALL reconstruction can improve the current effectiveness of ACL reconstruction surgery. We need further anatomical and biomechanical research on the ALL, which will help improve the level of reconstruction technology. At present, there is no literature indicating whether shared tendon graft reconstruction of the ACL and ALL has a better postoperative effect than the respective reconstruction of the ACL and ALL. However, in terms of preserving tendons and preventing tunnel convergence, shared tendon graft reconstruction of the ACL and ALL shows significant advantages. In terms of graft fixation sites, selecting ligamentous isometric points is crucial for the success of surgery, as the length of the ligaments during knee flexion and extension is non-isometric. If the graft is not fixed at an isometric point, excessive stretching or relaxation may occur, leading to surgical failure. However, scholars have not reached a consensus on the femoral and tibial isometric points of the ALL, which has led to significant differences in knee flexion angles when studying ligament fixation of knee joints due to inconsistent selection of isometric points. There have been reports of ligament fixation at angles such as 30°, 45°–60°, and 60°–90° (71). Lutz followed the practice of tightening and fixing the ligaments when the knee joint was almost fully extended and the tibia was in a neutral position (74). The ALL expert group also suggests that the ligaments should be fixed in an extended neutral position (75). While paying attention to both the function and the isomorphism of the ALL, we also need to strictly select based on indications before surgery. Physicians should pay special attention to the decisive indicators for diagnosing ALL injuries. When conducting axial shift tests with patients, it is recommended to combine manual examination and quantified indicator tools to avoid interference from subjective factors and to improve diagnostic accuracy. In addition, in terms of graft selection, the vast majority of publications in the literature report using the autologous iliotibial tendon and the hamstring tendon, but there are also allogeneic tendons. It has also been documented that patellar tendons can be used as grafts for ACL reconstruction, with no significant difference in long-term functional outcome compared with hamstrings. However, there is currently no literature suggesting that patellar tendons can be co-grafted with other tendons to reconstruct the ACL and ALL (76). Each of them has their own strengths and weaknesses. In terms of surgical methods, we divide ACL and ALL reconstruction using a shared bundle of grafts into three categories: single-bundle ACL reconstruction combined with single-bundle ALL reconstruction, double-bundle ACL reconstruction combined with single-bundle ALL reconstruction, and single-bundle ACL reconstruction combined with double-bundle ALL reconstruction. So far, no study has reported on which method is the optimal choice for surgery. In summary, shared tendon graft reconstruction of the ACL and ALL presents ideas and directions for further improving functional recovery in patients with ACL injury, and there are also many issues that need further research. We believe that with the further development of this practice, surgical techniques will continue to be optimized, enabling patients to return to social life more successfully and more quickly.
